# Supplementary material for: T cell fate following Salmonella infection is determined by a STING-IRF1 signaling axis in mice
Source: Commun Biol. 2019 Dec 11;2:464. doi: 10.1038/s42003-019-0701-2 (PMC6906324; doi:10.1038/s42003-019-0701-2)
Supplement: Supplementary file 1 — Description of Additional Supplementary Files [file 42003_2019_701_MOESM1_ESM.pdf]

Supplementary data 1

Related to Fig.4a, b.
RNA-seq analysis of BMDCs from wild-type, *Tmem173*$^{-/-}$, *Irf1*$^{-/-}$ and *Irf3/7*$^{-/-}$ mice after 18 hours of stimulation with c-di-GMP. Cuffdiff analysis, revealed a total of 4,588 genes that were found to be differentially expressed by more than twofold with a false discovery rate (FDR) cutoff of 0.05 between indicated groups.

Supplementary data 2

Related to Fig.4b.
1869 differentially expressed genes that were significantly upregulated more than twofold could further be differentiated into clusters that were dependent on *Irf1* and/or *Irf3/7* distinguishing 6 signatures that were dependent on *Irf1* or *Irf3/7* alone or regulated by both *Irf1* and *Irf3/7* together.
