# Supplementary material for: T cell fate following Salmonella infection is determined by a STING-IRF1 signaling axis in mice
Source: Commun Biol. 2019 Dec 11;2:464. doi: 10.1038/s42003-019-0701-2 (PMC6906324; doi:10.1038/s42003-019-0701-2)
Supplement: Supplementary file 4 — Supplementary Materials [file 42003_2019_701_MOESM4_ESM.pdf]

Supplementary Materials

Supplementary Figure 1. Supporting evidence for IRF1, IRF3 and IFNAR dependent gene expression.

(**a**) qRT-PCR analysis of *Il12rb1* and *Il12rb2* mRNA expression in BMDCs isolated from wild-type and *Irf1*$^{-/-}$mice after 4- and 18-hours stimulation with 25µg/ml of c-di-GMP. n=4. * $p < 0.01$.

(**b-d**) qRT-PCR analysis of the mRNA expression of indicated genes in BMDCs from wild-type, *Irf1*$^{-/-}$, *Irf3*$^{-/-}$;*Ifnar*$^{-/-}$ mice after 4 and 18 hours stimulation with 25µg/ml of c-di-GMP. Data shows mean ± SEM of two independent experiments. N=4 * $p < 0.01$. NS, not significant. Two-way ANOVA with a Tukey's multiple comparisons test (a-d).

Supplementary Figure 1

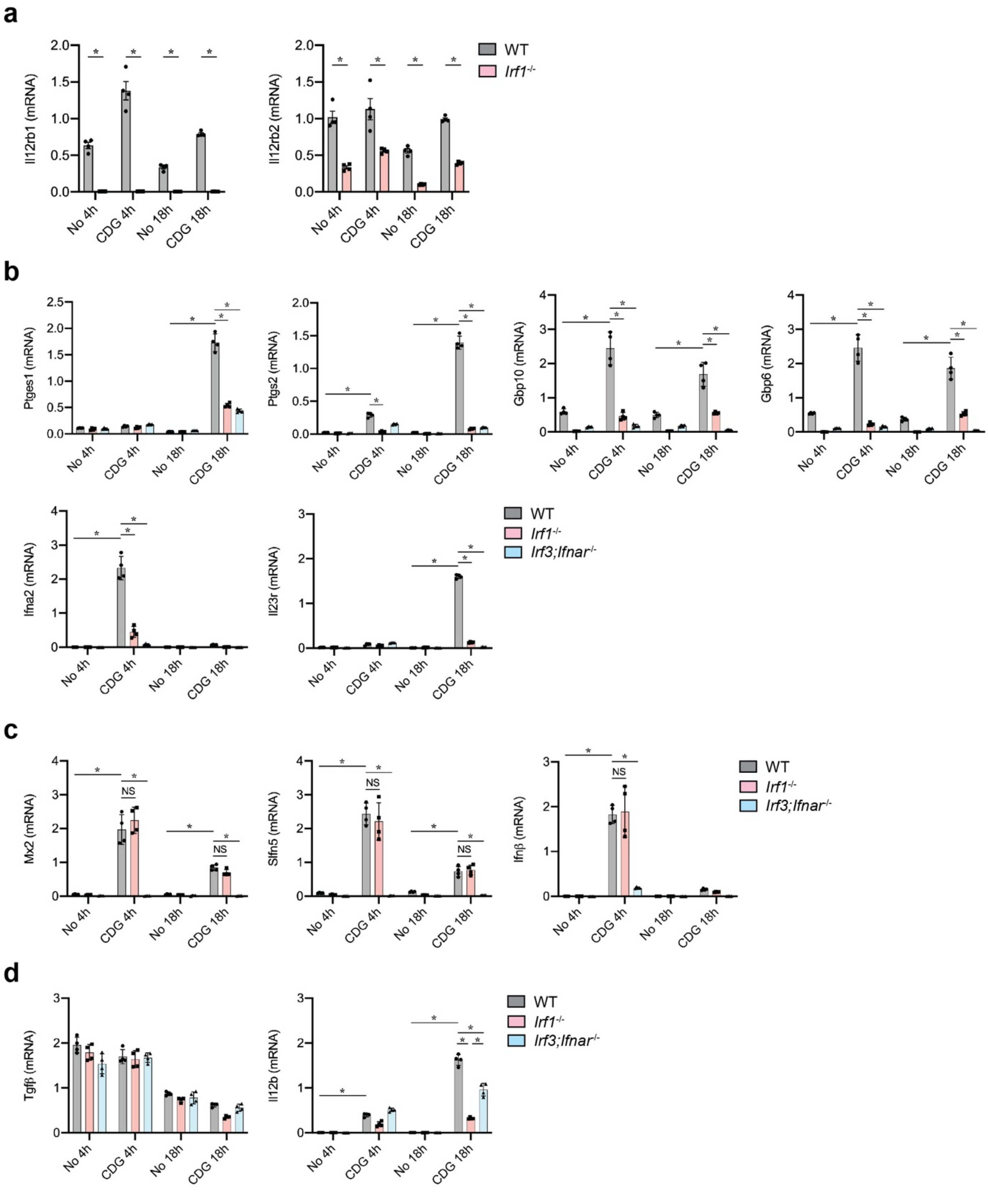

Supplementary Figure 2. Inflammasome activation in BMDCs from wild-type, Tmem173$^{-/-}$ and Irf1$^{-/-}$ mice after LPS and ATP stimulation.

(**a**) IL-1α and IL-1β protein expression in supernatants of LPS-primed BMDCs isolated from wild-type, *Tmem173*$^{-/-}$ and *Irf1*$^{-/-}$ mice after stimulation with ATP. n=6. * $p < 0.001$. Two-way ANOVA with a Tukey's multiple comparisons test.

Supplementary Figure 2

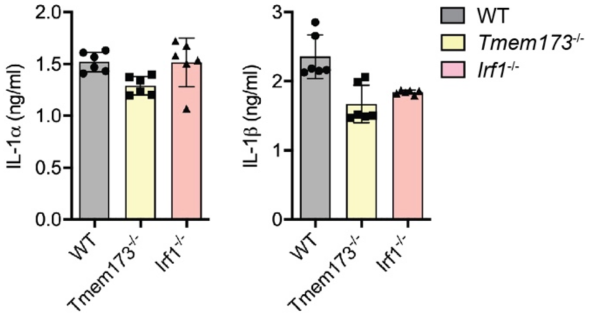

Supplementary Figure 3. Cluster analysis of wild-type, IRF1 and IRF3/7 dependent gene expression signatures in response to c-di-GMP stimulation.

Heat maps showing genes contained in the clusters aggregated in Fig.4c that dependent on the presence of IRF1 and IRF3/7 (cluster 3), that are upregulated in the absence of IRF3/7 (cluster 4) or dependent on the presence of IRF3/7 (cluster 5). Key regulators are indicated in red.

Supplementary Figure 3

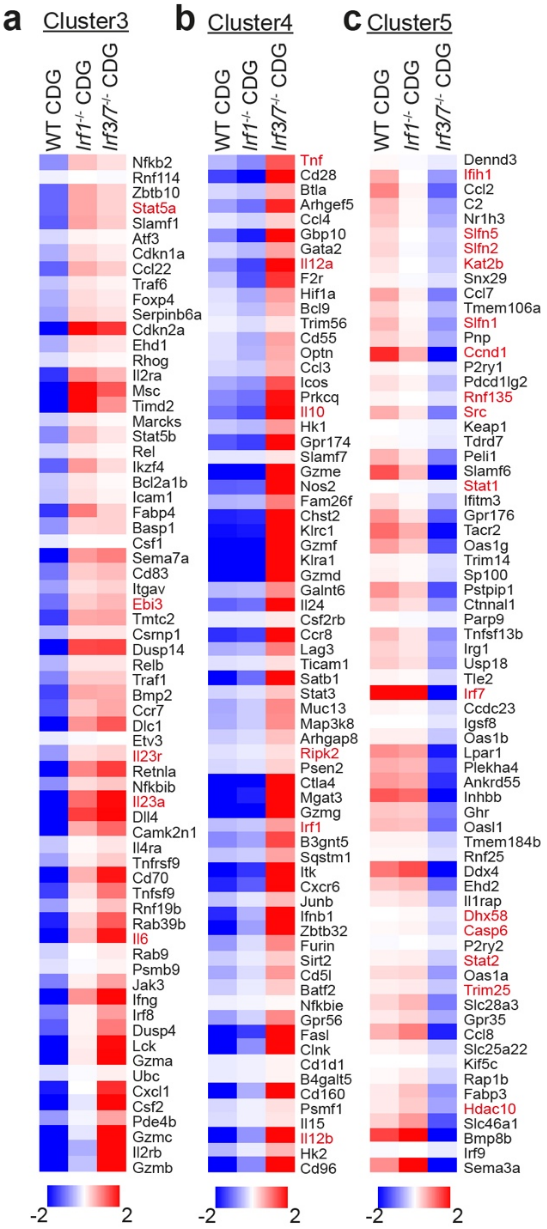

Supplementary Figure 4. IL-1β re-establishes IFNγ but not IL-17A expression in Tmem173$^{-/-}$ mice.

Il17a, and *Ifnγ* expression in CD4^{+} T cells from *Tmem173*$^{-/-}$ verse wild-type mice. Wild-type and *Tmem173*$^{-/-}$ mice were immunized with OVA plus c-di-GMP *i.p.* and injected with recombinant mouse IL-1β on day 1 and day 3 before being sacrificed on day 5. n=4. *** $p < 0.001$. *P*-values, *** Two-way ANOVA with a Tukey's multiple comparisons test.

Supplementary Figure 4.

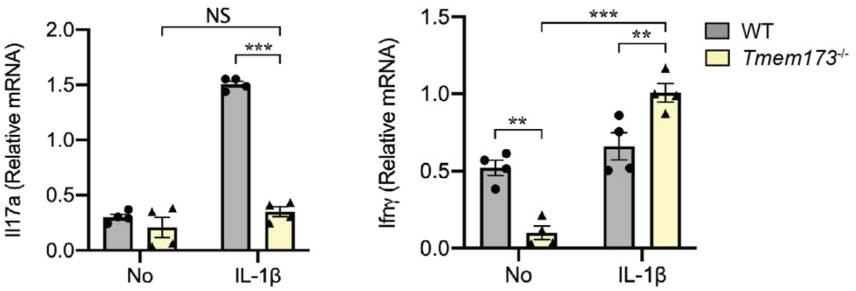

Supplementary Figure 5. All blot images of Figure 2 and Figure 5.

Supplementary Figure 5. All blot images of Figure 2 and Figure 5.

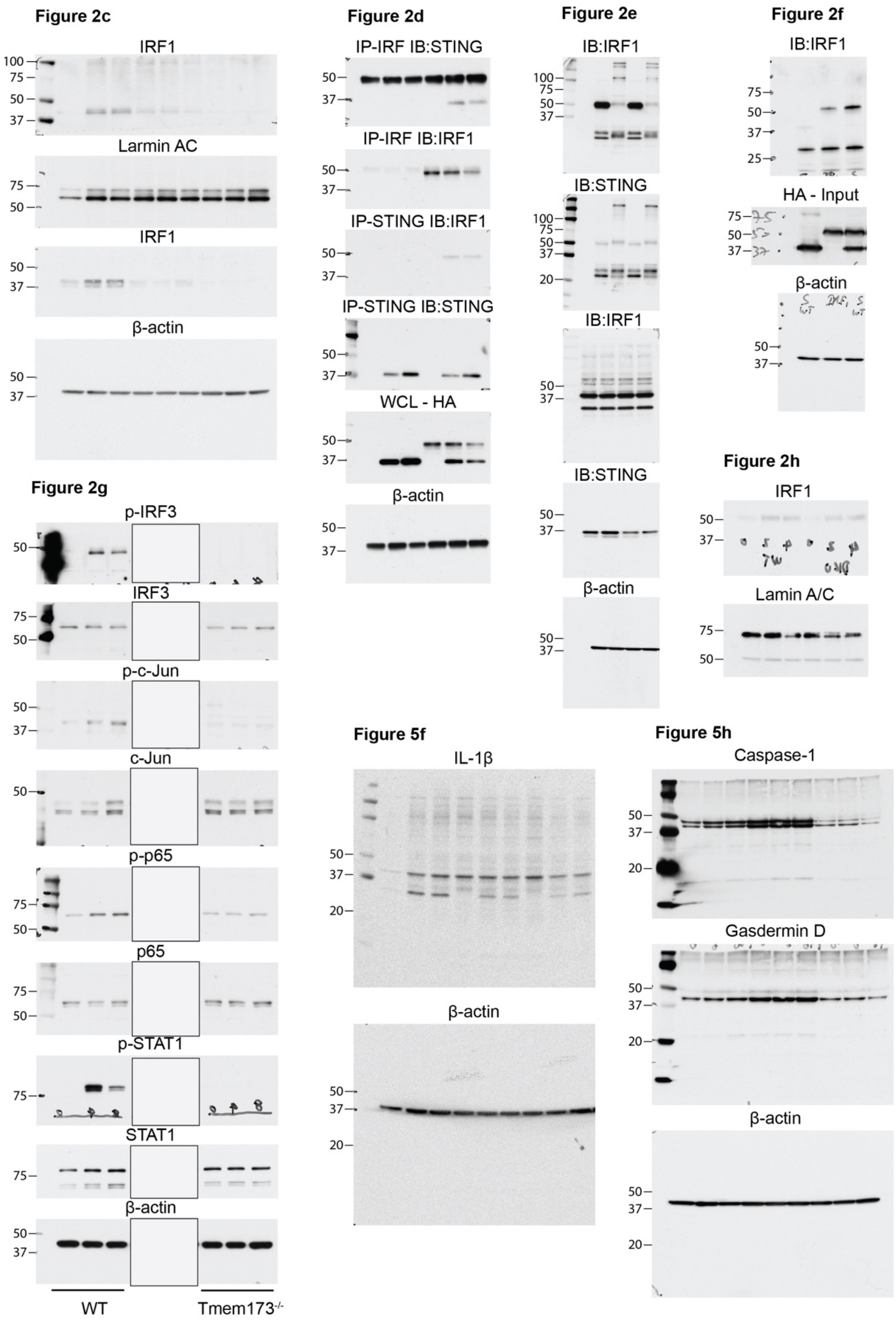

Supplementary Table 1. Primers sequences used in this study.

Genes	Forward 5' to 3'	Reverse 5' to 3'
18S	GTAACCCGTTGAACCCCATT	CCATCCAATCGGTAGTAGCG
Gapdh	TGACCTCAACTACATGGTCTACA	CTTCCCATTCTCGGCCTTG
Casp1	TACACGTCTTGCCCTCATTATC	CTCCAGCAGCAACTTCATTTC
Gbp10	CCGTACTGGGAAGTCCTATTTG	CAGATGCCCTTGGTTTGAGA
Gbp6	GTCTGGACTGTTCGGGATTT	GCTTGCATTCTGGGTTTGTC
Gsdmd	CTCCTGTCAGATGGGATTGATG	CCATTTCCAAGCTCTCCAGTT
Ifna2	CTTTCCTCGTGATGCTGATAGT	CCTTCAAGGCCCTCTTGTT
Il1α	GAAGAAGAGACGGCTGAGTTT	TCACTCTGGTAGGTGTAAGGT
Il1β	GCAACTGTTCCTGAACTCAACT	ATCTTTTGGGGTCCGTCAACT
IL2	AGCAGCTGTTGATGGACCTA	CGCAGAGGTCCAAGTTCAT
IL4	GGTCTCAACCCCCAGCTAGT	GCCGATGATCTCTCTCAAGTGAT
Il6	TAGTCCTTCCTACCCCAATTTCC	TTGGTCCTTAGCCACTCCTTC
Il12a	AGACATCACACGGGACCAAAC	CCAGGCAACTCTCGTTCTTGT
Il12b	TGGTTTGCCATCGTTTTGCTG	ACAGGTGAGGTTCACTGTTTCT
Il12rb1	CTACAGGGTTTCCAAGACAGAC	GTATGGTTCGGAGGGACAAAG
Il12rb2	AGAGAATGCTCATTGGCACTTC	AACTGGGATAATGTGAACAGCC
Il17a	TGAGCTTCCCAGATCACAGA	TCCAGAAGGCCCTCAGACTA
Il17f	TGCTACTGTTGATGTTGGGAC	AATGCCCTGGTTTTGGTTGAA
Il18	ACCTTCCAAATCACTTCCTCTT	GTCTGATTCCAGGTCTCCATTT
Il21	CGCCTCCTGATTAGACTTCG	CAGGGTTTGATGGCTTGAGT
Il22	ATGAGTTTTTCCCTTATGGGGAC	GCTGGAAGTTGGACACCTCAA
Il23a	CAGCAGCTCTCTCGGAATCTC	TGGATACGGGGCACATTATTTTT
Il23r	TCAGTGCTACAATCTTCAGAGGACA	GCCAAGAAGACCATTCCCGA
Il27	AGCCTGTTGCTGCTACCCTTGC	GTGGACATAGCCCTGAACCTCA
Il33	TCCAACTCCAAGATTTCCCCG	CATGCAGTAGACATGGCAGAA
Il36α	GCAGCTCAGAAACAACATCAC	AGGATCCACACACGACTACTA
Ifnβ1	GGCAGATGTCCTCAACTGCTC	GACCACCATCCAGGCGTAG
Ifnγ	ACAGCAAGGCGAAAAAGGATG	TGGTGGACCACTCGGATGA
Irf1	CAGAGGAAAGAGAGAAAGTCC	CACACGGTGACAGTGCTGG
Irf3	GGCTTGTGATGGTCAAGGTT	TGGGGCTCAGATATTTCCAG
Irf7	CACCCCCATCTTCGACTTCA	CCAAAACCCAGGTAGATGGTGTA
Mx2	GAATTACCAGGGTGGCTGTAG	CAGGTTGATGGTCTCCTGTTT
Ptgs1	GGATACTGGCTCTGGGAATTTG	GTAGTCATGCGCTGAGTTGTAG

Ptgs2	CGGACTGGATTCTATGGTGAAA	CTTGAAGTGGGTCAGGATGTAG
Ptges1	CCACACTCCCTCTTAACCATAAA	GCCAGAATTGTAGGTAGGTCTG
RoRγt	GCTCCATATTTGACTTTTCCCACT	GATGTTCCACTCTCCTCTTCTCTTG
Slfn5	AAGGGAGGAAATGGATACCACA	CTCAACCCTGACCACTCCG
Tgfβ1	CCACCTGCAAGACCATCGAC	CTGGCGAGCCTTAGTTTGGAC
