# Supplementary material for: T cell fate following Salmonella infection is determined by a STING-IRF1 signaling axis in mice
Source: Commun Biol. 2019 Dec 11;2:464. doi: 10.1038/s42003-019-0701-2 (PMC6906324; doi:10.1038/s42003-019-0701-2)
Supplement: Supplementary file 5 — Reporting Summary [file 42003_2019_701_MOESM5_ESM.pdf]

nature research

Corresponding author(s): Hans-Christian Reinecker

Last updated by author(s): Nov 6, 2019

Reporting Summary

Nature Research wishes to improve the reproducibility of the work that we publish. This form provides structure for consistency and transparency in reporting. For further information on Nature Research policies, see Authors & Referees and the Editorial Policy Checklist.

Statistics

For all statistical analyses, confirm that the following items are present in the figure legend, table legend, main text, or Methods section.

n/a	Confirmed	
☐	☒	The exact sample size (*n*) for each experimental group/condition, given as a discrete number and unit of measurement
☐	☒	A statement on whether measurements were taken from distinct samples or whether the same sample was measured repeatedly
☐	☒	The statistical test(s) used AND whether they are one- or two-sided<br>*Only common tests should be described solely by name; describe more complex techniques in the Methods section.*
☒	☐	A description of all covariates tested
☐	☒	A description of any assumptions or corrections, such as tests of normality and adjustment for multiple comparisons
☐	☒	A full description of the statistical parameters including central tendency (e.g. means) or other basic estimates (e.g. regression coefficient) AND variation (e.g. standard deviation) or associated estimates of uncertainty (e.g. confidence intervals)
☐	☒	For null hypothesis testing, the test statistic (e.g. *F*, *t*, *r*) with confidence intervals, effect sizes, degrees of freedom and *P* value noted<br>*Give P values as exact values whenever suitable.*
☒	☐	For Bayesian analysis, information on the choice of priors and Markov chain Monte Carlo settings
☐	☒	For hierarchical and complex designs, identification of the appropriate level for tests and full reporting of outcomes
☒	☐	Estimates of effect sizes (e.g. Cohen's *d*, Pearson's *r*), indicating how they were calculated

Our web collection on statistics for biologists contains articles on many of the points above.

Software and code

Policy information about availability of computer code

Data collection	Flow cytometry data was collected using FACS Diva (BD biosciences)
Data analysis	RNA-seq data were analyzed with DNAnexus (Tophat, Cuffdiff) and Seqmonk software version 1.41.0.<br>Statistical analysis was performed using GraphPadPrism version 6.07. Flow cytometry data was analyzed using Flowjo version 10.1r5.

For manuscripts utilizing custom algorithms or software that are central to the research but not yet described in published literature, software must be made available to editors/reviewers. We strongly encourage code deposition in a community repository (e.g. GitHub). See the Nature Research guidelines for submitting code & software for further information.

Data

Policy information about availability of data

All manuscripts must include a data availability statement. This statement should provide the following information, where applicable:

- Accession codes, unique identifiers, or web links for publicly available datasets
- A list of figures that have associated raw data
- A description of any restrictions on data availability

The RNA-seq data have been deposited to the Gene Expression Omnibus under the accession no GSE137428. Available at https://www.ncbi.nlm.nih.gov/geo/query/acc.cgi?acc=GSE137428. The source data that support the findings of this study are available from the corresponding author upon request.

Field-specific reporting

Please select the one below that is the best fit for your research. If you are not sure, read the appropriate sections before making your selection.

☒ Life sciences ☐ Behavioural & social sciences ☐ Ecological, evolutionary & environmental sciences

For a reference copy of the document with all sections, see nature.com/documents/nr-reporting-summary-flat.pdf

Life sciences study design

All studies must disclose on these points even when the disclosure is negative.

Sample size	The cell samples were collected with two or three duplicate and performed with two or three independent experiments. The samples from animal were collected from three or four mice every group and performed with two or three independent experiments.
Data exclusions	There were no excluded samples or data from the analysis in this study.
Replication	Each experiment was repeated at least twice as indicated in the figure legend. All experimental data was reproducible with similar observation in each attempt.
Randomization	We randomly choose mice from the same or different litermates for each experiment group and also randomly chose the control mice with same sex and similar date birth.
Blinding	N/A No blinding method was applied.

Reporting for specific materials, systems and methods

We require information from authors about some types of materials, experimental systems and methods used in many studies. Here, indicate whether each material, system or method listed is relevant to your study. If you are not sure if a list item applies to your research, read the appropriate section before selecting a response.

Materials & experimental systems

n/a	Involved in the study	
☐	☒	Antibodies
☐	☒	Eukaryotic cell lines
☒	☐	Palaeontology
☐	☒	Animals and other organisms
☒	☐	Human research participants
☒	☐	Clinical data

Methods

n/a	Involved in the study	
☒	☐	ChIP-seq
☐	☒	Flow cytometry
☒	☐	MRI-based neuroimaging

Antibodies

Antibodies used	CD103 (M290), CD11c (HL3), CD3 (145-2C11), CD4 (GK1.5) and MHC II (M5/114. 15.2) were purchased from BD biosciences. CD11b (M1/70) and CD45 (30-F11) were purchased from Biolegend. IRF1 (D5E4), STING (D2P2F), Lamin A/C (4C11), IRF3 (D6I4C), Phospho-IRF3 (D601M), c-Jun (60AB), Phospho (S63)-c-Jun (D46G9), p65(D14E12), Phospho (S536)-p65 (93hH1), STAT1 (Cat No.9172), Phospho (Y701)-STAT1 (58D6) IL-1beta (3A6) and beta-actin (8h10d10) were obtained from Cell Signaling. Caspase 1(Casper-1), Gsdmd (IN110) and HRP-conjugated anti-Guinea pig IgG (AG-29B-0008E)were obtained from Adipogen. HRP-conjugated anti-rabbit IgG (NA934), HRP-conjugated mouse IgG (NA931) were obtained from GE Healthcare .
Validation	All antibodies used in this study were obtained from commercial sources and validated according to manufacturers's instruction. Additionally, most immuno blot antibodies were validated as demonstrated in our previous works. FACS antibodies were validated using titeration before use.

Eukaryotic cell lines

Policy information about cell lines

Cell line source(s)	HEK293T cells from ATCC
Authentication	The cell line were purchased from ATCC and authenticated by the vendor.
Mycoplasma contamination	The cell line were tested Mycoplasma negative.

Commonly misidentified lines (See ICLAC register)	sidentified cell line were used in this study.

Animals and other organisms

Policy information about studies involving animals; ARRIVE guidelines recommended for reporting animal research

Laboratory animals	*For laboratory animals, report species, strain, sex and age OR state that the study did not involve laboratory animals.*
Wild animals	C57BL/6, Tmem173-/-, IRF1-/-, IRF3/7-/-, IRF7-/-, IRF3/IFNAR-/-, IL-17A-GFP, Cx3cr1-GFP, Zbta46-GFP, CAG::KikGR and OT-II TCR Tg mice were used in this study. IL-17A-GFP mice and CAG::KikGR mice were crossed with Tmem173-/- mice. Genotypes of those mice were verified by PCR analysis. 6-12 week old male and female mice were analyzed. All the animal procedures were conducted in accordance with approved protocols by the Animal Care and Use Committee at Massachusetts General Hospital and Harvard Medical School.
Field-collected samples	N/A
Ethics oversight	N/A

Note that full information on the approval of the study protocol must also be provided in the manuscript.

Flow Cytometry

Plots

Confirm that:

☒ The axis labels state the marker and fluorochrome used (e.g. CD4-FITC).

☒ The axis scales are clearly visible. Include numbers along axes only for bottom left plot of group (a 'group' is an analysis of identical markers).

☒ All plots are contour plots with outliers or pseudocolor plots.

☒ A numerical value for number of cells or percentage (with statistics) is provided.

Methodology

Sample preparation	The cells were collected from bone-marrow, spleen, draining lymph node and laminar propria cells from small intestine, filtered with strainer, removed RBC using ACK buffer, stained and analyzed for flow cytometry. Laminar propria cells of small intestine were prepared by Collagenase (Liberase TL, Roche) digestion. Primary dendritic cells from Peyer's patch and MLN were isolated by Collagenase A digestion.
Instrument	BD LSR II, BD FACS Aria III were used for data collection.
Software	FACS Diva and FlowJo software were used.
Cell population abundance	Cell population abundance was described in the manuscript.
Gating strategy	Gating strategy was described in the manuscript.

☐ Tick this box to confirm that a figure exemplifying the gating strategy is provided in the Supplementary Information.
